# Supplementary material for: Tropical volcanoes synchronize eastern Canada with Northern Hemisphere millennial temperature variability
Source: Nat Commun. 2022 Aug 26;13:5042. doi: 10.1038/s41467-022-32682-6 (PMC9418434; doi:10.1038/s41467-022-32682-6)
Supplement: Supplementary file 1 — Supplementary Information [file 41467_2022_32682_MOESM1_ESM.pdf]

Supplementary Information for

Tropical volcanoes synchronize eastern Canada with Northern Hemisphere millennial temperature variability

Feng Wang[1]*[#], Dominique Arseneault[1], Étienne Boucher[2], Fabio Gennaretti[3], Shulong Yu[4], Tongwen Zhang[4]

[1]Département de Biologie, Chimie et Géographie, Centre d'Études Nordiques, Université du Québec à Rimouski, Rimouski, G5L 3A1, Canada
[2]Département de Géographie, GEOTOP, and Centre d'Études Nordiques, Université du Québec à Montréal, Montréal, H2X 3R9, Canada
[3]Institut de Recherche sur les Forêts, Groupe de Recherche en Écologie de la MRC-Abitibi, Centre d'Étude de la Forêt, Université du Québec en Abitibi-Témiscamingue, Amos, J9T 2L8, Canada
[4]Xinjiang Key Laboratory of Tree-Ring Ecology, Key Laboratory of Tree-Ring Physical and Chemical Research, Institute of Desert Meteorology, China Meteorological Administration, Urumqi, 830002, China

*Corresponding author: Feng Wang
Email: feng.wang@uqar.ca

#: Current address: Centre Eau Terre Environnement, Institut National de la Recherche Scientifique, Québec, G1K 9A9, Canada.

Inventory

Supplementary Methods 1–4.
Supplementary Figures 1–14.
Supplementary Tables 1–10.

Supplementary Methods

Supplementary Method 1. Validation of Quex maximum latewood density (MXD) data.

The Quex MXD dataset was validated before being included in our density network and some problems were found. Firstly, the site location must be corrected by ~300 km from the town of Kuujjuarapik (55.33ºN, 77.83ºW) to the region of the Boniface River (57.75ºN, 76.17ºW). The Boniface River area is a region of relict forests and the only known location in northern Quebec where > 500 years of long black spruce tree-ring chronologies can be developed from dried spruce trunk at the soil surface[1,2]. An additional indication of the erroneous location is that the site Quex is labeled as "Bonif historic" in the NOAA International Tree Ring Data Bank, coinciding with Boniface River. In order to confirm the origin of Quex, the dataset was correlated over the period 1901–1988 CE against a black spruce ring-width network comprising 60 sites across the Quebec-Labrador Peninsula. Tree-ring width (TRW) data were used because they are more abundant and display a smaller spatial domain than does MXD[3]. Considering that tree replications varied among sites, we standardized ring width as ratios using individual age-dependent spline with the signal-free approach (a suitable method for standardizing living black spruce ring-width series[4]) at each site and used the first-differenced data to amplify interannual variability. The Pearson's correlation coefficients clearly show a strengthening relation with the Quex ring width toward the Boniface River region, away from the wrong location of Kuujjuarapik (Supplementary Fig. 13a). A similar eccentric location is also evidenced by the correlation field of the raw (not first-differenced) Quex ring-width chronology against gridded CRU data (Supplementary Fig. 13b).

Furthermore, there are several zero values in some of the MXD series of Quex that were likely due to very narrow rings or measurement errors, because missing rings are extremely rare in black spruce in northern Quebec. We accordingly replaced these zero values with the available density data from a second radius of the same tree ID in the corresponding year. A missing value was only kept if there was no density measurement from the second radius. In addition, cross-dating of one core series (ID: 858181) was corrected because of a dating error. Core series were then averaged by tree for further analyses. We also estimated a pith offset of 2 rings (missing metadata; the modal value in our own data) for each tree series for the subsequent standardization.

Supplementary Method 2. Comparison of standardization methods.

To optimize temperature signals in our final reconstructions, we compared three methods to standardize MXD data at the four sites, including the conventional regional curve standardization (RCS)[5], RCS with signal-free approach (sfRCS)[6], and regionally constrained individual signal-free standardization (RSFi)[7]. Chronologies were calculated based on ratios and residuals plus power-transformation (hereafter named ratio chronology and residual chronology, respectively) for the three methods using the RCSigfree program (https://www.ldeo.columbia.edu/tree-ring-laboratory/resources/software; last access: 18 July, 2022). For all methods, we used age-dependent spline smoothing[8] with an initial stiffness of 2 years to estimate the growth trends of the MXD series, and chronologies were calculated using Tukey's bi-weight robust mean along with the variance stabilization procedure[9,10].

We calculated Pearson's correlation coefficients among local chronologies, and between each chronology and local May–August (MJJA) temperatures averaged from monthly data of the four gridded cells closest to each site (CRU dataset). This analysis shows that the RSFi method in general resulted in stronger correlations among sites and with the (raw and 10-year low-pass) local temperature target (Supplementary Table 10), and could correct for the "divergent trends" in recent decades that are evident with other methods, in particular for the site L135 (Supplementary Fig. 14). In general, the ratio chronologies showed slightly higher correlations (among sites and with

MJJA temperatures) than the residual chronologies across standardization methods, although this trend was not evident for the RSFi method (Supplementary Table 10).

The skill of the RSFi ratio and residual chronologies was further assessed via temperature reconstructions using a time-efficient linear scaling reconstruction approach[11] that yields similar results to the linear Bayesian method[12] (Supplementary Fig. 10). For this assessment, we used RSFi chronologies at the four sites. The scaling method forced each local series to have the same mean and variance as those of the regional MJJA temperature target over the 1905–2006 time period (1905–1989 CE for site Quex). The scaled chronologies were then arithmetically averaged and scaled again with respect to the temperature target to form a scaled reconstruction. In agreement with comparisons of most sites (Supplementary Table 10), the regional MJJA temperatures were also better correlated when using the ratio-based reconstruction (Supplementary Fig. 10). Consequently, RSFi ratio chronologies were chosen for our final temperature reconstructions.

Supplementary Method 3. RSFi standardization.

The regionally constrained individual signal-free standardization (RSFi) method[7] combines the individual signal-free standardization with the signal-free RCS method. At each site, we first used the RCSigfree program to standardize MXD data using the individual signal-free age-dependent spline smoothing to generate individual growth trends (curves) for each individual series. The sfRCS was also applied to generate a signal-free regional curve. Briefly, each individual growth curve was aligned with the sfRCS regional curve according to cambial age, and was adjusted to the same mean value as that of the regional curve for the same cambial age interval. The mean-adjusted individual growth curves were then used to re-standardize raw MXD series. This procedure is efficient for removing irregular growth trends due to atypic growth rates and disturbances in different time periods, while also preserving the low-frequency domain that would be lost when using only individual-based standardization. Chronologies were finally calculated using Tukey's bi-weight robust mean along with the variance stabilization procedure[9,10].

Supplementary Method 4. Bayesian reconstruction.

The linear Bayesian approach used for our summer temperature reconstructions was originally designed to perform a multi-proxy reconstruction[12]. Here, we use this approach with multiple proxy series. The posterior density of the reconstructed climate (c_i) at year i, given by N proxy series $\boldsymbol{D}_i = [d_{1,i}, d_{2,i}, \ldots, d_{N,i}]$ and observed climate data (C_{cal}) of the same time scale, can be written as:

$$p(c_i|\boldsymbol{D}_i, \boldsymbol{D}_{cal}, C_{cal}) \propto \left[\prod_{k=1}^{N} \int p(d_{k,i}|c_i, \tau) p(d_{k,cal}|C_{cal}, \tau) p(\tau)\, d\tau\right] p(c_i). \quad \text{(Eq.1)}$$

where $d_{k,\ cal}$ is the data of the kth series over the calibration period, and τ is a set of parameters $[\alpha, \beta, \sigma]$ defining the linear relationship between each proxy (i.e., MXD) series and the climate data with normally distributed errors:

$$p(d_{k,i}|c_i, \tau) \equiv N(d_{k,i};\ \mu = \alpha c_i + \beta, \sigma). \quad \text{(Eq.2)}$$

Equation (2) can be solved using $d_{k,cal}$ and C_{cal} (i.e., $p(d_{k,cal}|C_{cal}, \tau)$), the proxy and climate data during the calibration period, and Markov Chain Monte Carlo sampling with Metropolis–Hastings steps.

In equation (1) the term $p(c_i)$ is a prior distribution of climate (i.e., given by the linear scaling MXD-based reconstruction in our cases, see below) and was assumed to be normally distributed:

$$p(c_i) \equiv N(c_i;\, \mu_c,\, \sigma_c). \tag{Eq.3}$$

For D-STREC and 3P-STREC, α, β, and σ were assumed to be a uniform prior in $sin(tan^{-1}(\alpha))$, a uniform prior, and a Jeffreys prior, respectively. We ran three parallel chains and obtained a total of 90,000 draws (after discarding 5000 burn-in times for each chain) to generate posteriors for these three parameters. Parameters for $p(c_i)$ were derived from the MXD-based temperature reconstruction using the linear scaling method (Supplementary Fig. 10a). In order to reconstruct the high-frequency (HF) components of 3P-STREC, the scaled temperature reconstruction (prior) was also decomposed using a 9-year-triangular filter to generate HF priors.

Supplementary Figures

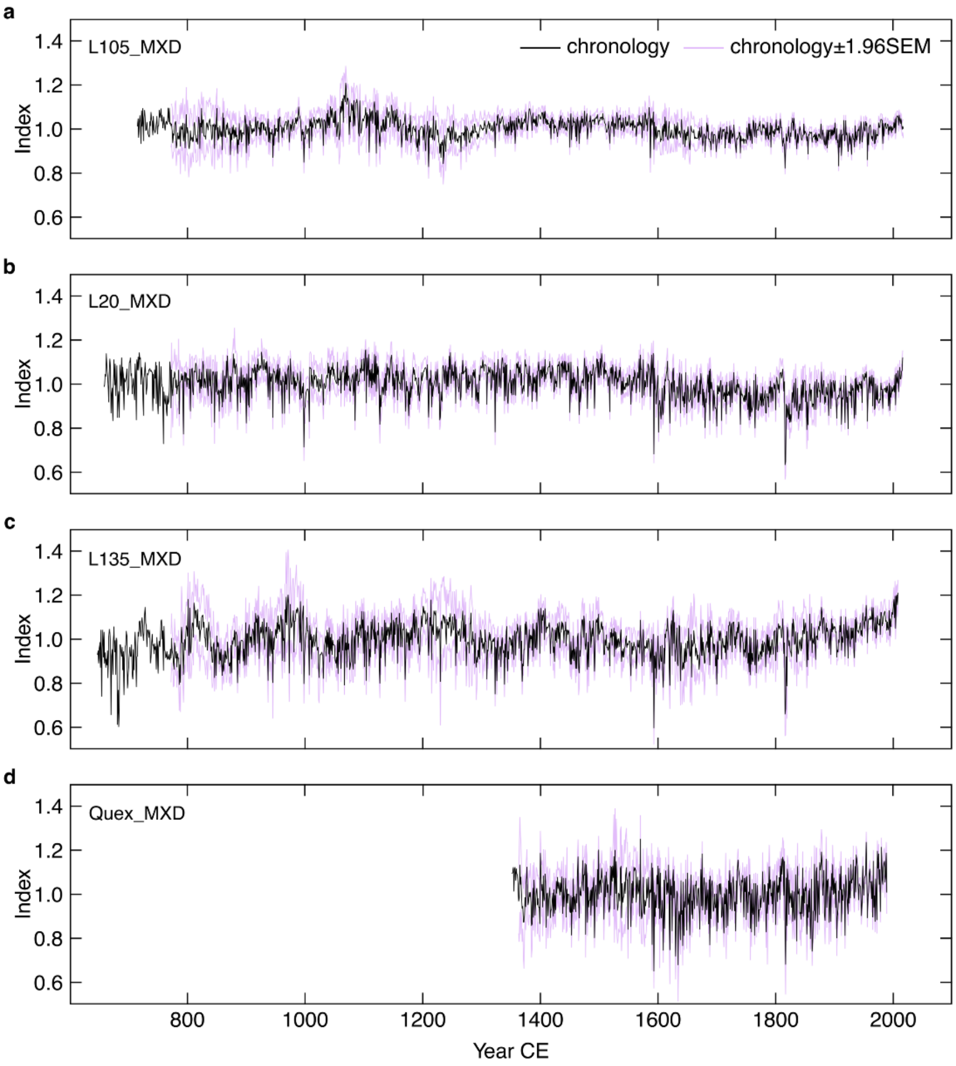

Supplementary Fig. 1. Local maximum latewood density (MXD) chronologies standardized using the regionally constrained individual signal-free standardization method (Supplementary Method 3). **a–d** Chronologies for sites L105, L20, L135, and Quex, respectively. Purple curves represent the ± 1.96 × standard error of mean (SEM) of available MXD measurements.

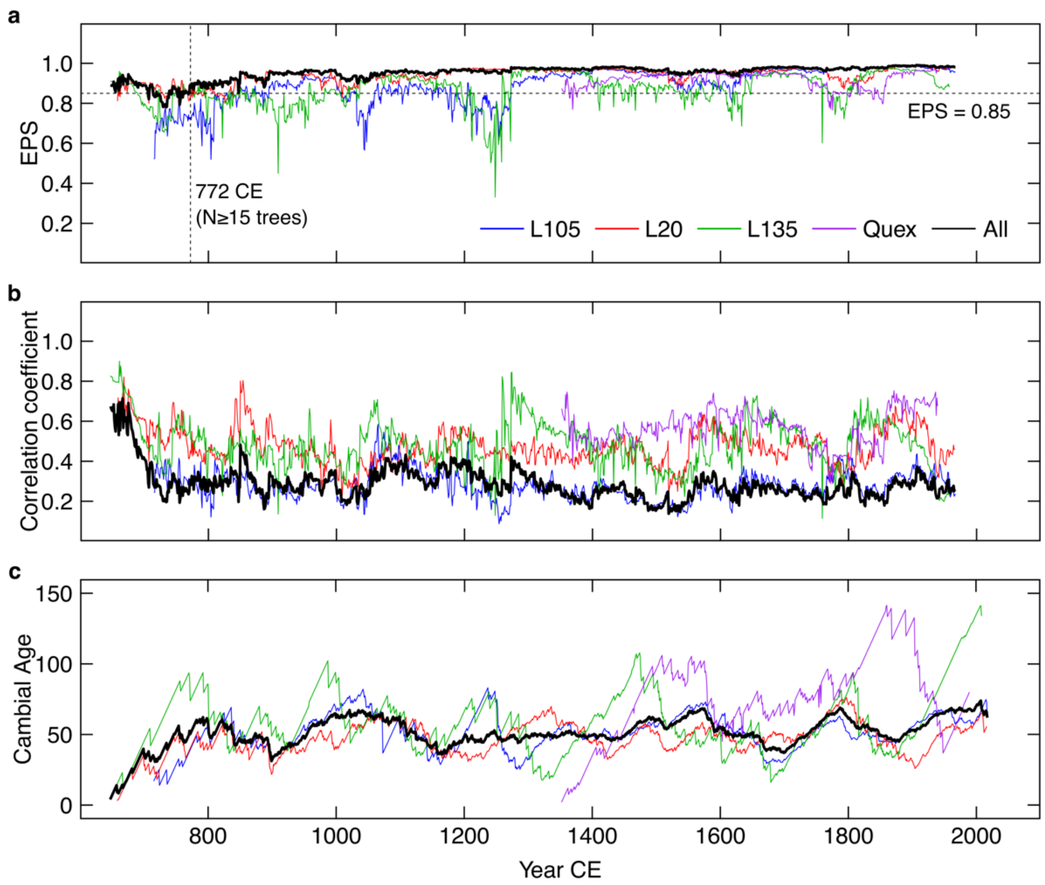

Supplementary Fig. 2. Summary statistics of local and regional maximum latewood density (MXD) series. **a** 51-year moving expressed population signal (EPS) values[13] aligned by the first year of each window. The regional EPS is consistently above 0.85 since 772 CE. **b** 51-year moving rbar statistics. **c** Mean cambial age. Mean cambial age of Quex dataset was estimated using a pith offset of 2 years for each tree series. Thick black curves show the statistics calculated using all MXD data from the four sites.

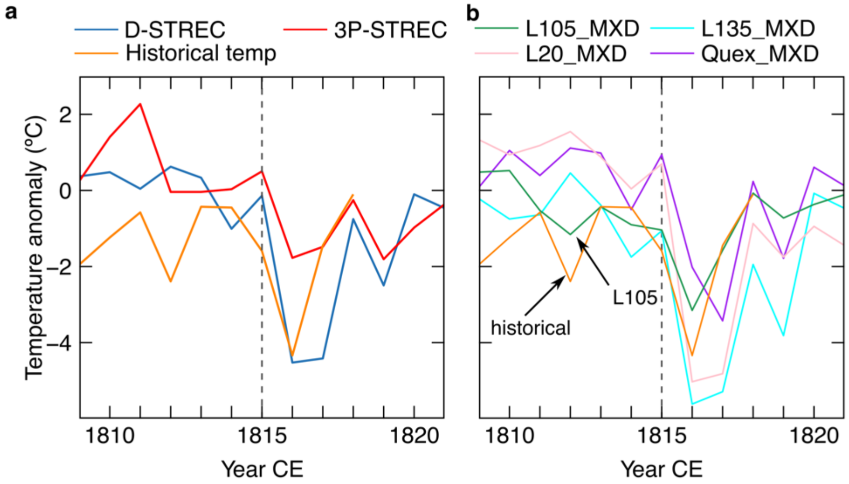

Supplementary Fig. 3. Tambora (1815 CE; dashed vertical line) cooling recorded in historical May–August temperatures versus two temperature reconstructions (**a**) and maximum latewood density (MXD) chronologies at four sites (**b**). L105 is the southernmost and closest to the Saint Lawrence Valley where the historical record was developed. L105 MXD chronology shows a consistent pattern from 1815–1817 CE compared to the historical data. The historical and reconstructed temperature series are shown as anomalies with respect to 1905–2006 CE. Local MXD chronologies are scaled relative to the historical temperature data over the 1905–2006 period (1905–1989 CE for the Quex chronology).

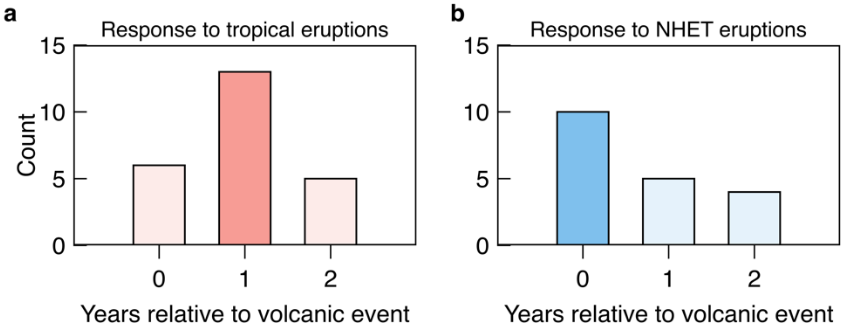

Supplementary Fig. 4. Timing of cooling maxima after tropical (**a**) and Northern Hemisphere extratropical (NHET, **b**) eruptions in D-STREC. Analyses are based on all the tropical and NHET eruptions listed in Supplementary Table 7.

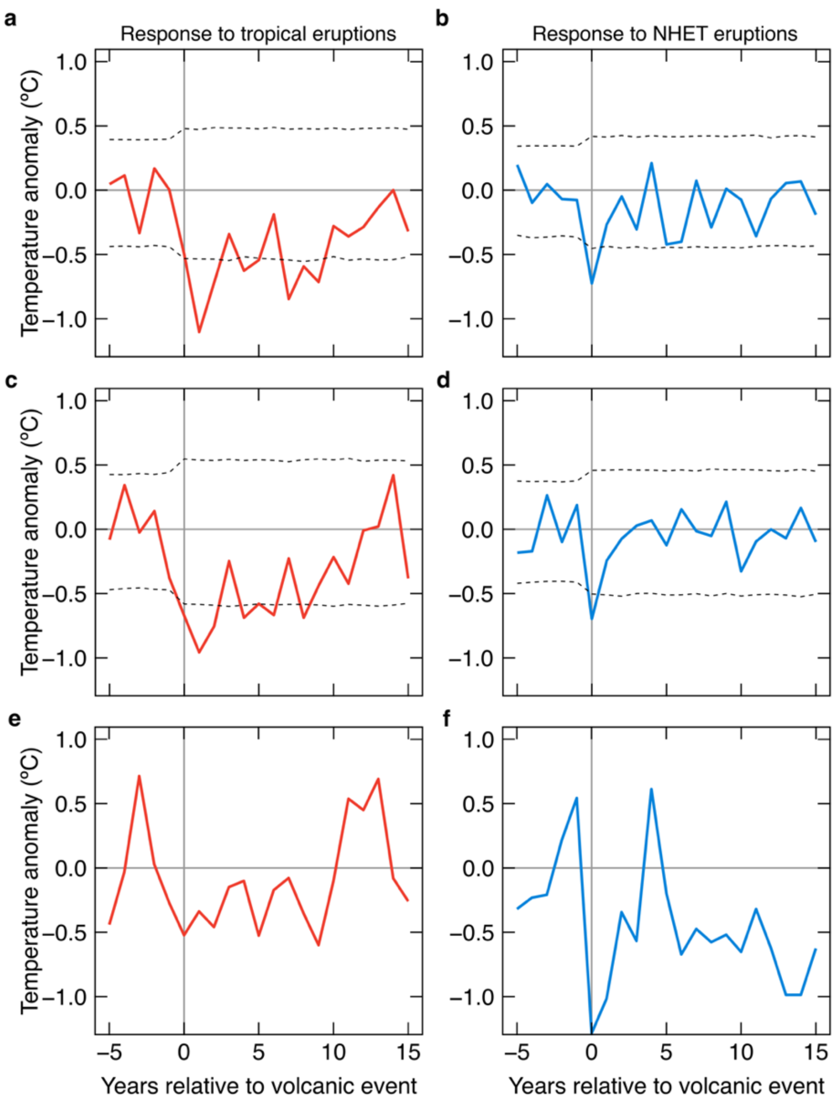

Supplementary Fig. 5. Validation of volcanic responses. **a** Superposed epoch analysis (SEA) of D-STREC using identified tropical eruptions (1257, 1586, 1593, 1600, 1641, 1673, 1815, 1831, 1835, 1862, 1883, 1903, 1963, and 1982 CE). **b** Same as **a**, but using identified Northern Hemisphere extratropical (NHET) eruptions (1210, 1477, 1480, 1510, 1668, 1708, 1740, 1756, 1766, 1783, 1857, 1873, 1907, and 1912 CE). **c** Same as **a**, but using tropical eruptions not interfered by other events (all events with stratospheric aerosol optical depth at 550 nm over 30–90ºN ($SAOD_{NHET}$) ≥ 0.03 provided by the eVovl2k plus CMIP6 dataset, including those not retained for SEA) within ± 9 years (1170, 1190, 1229, 1275, 1285, 1883, and 1963 CE). **d** Same as **c**, but using NHET eruptions not interfered by other events (1180, 1210, 1328, 1510, 1708, 1730, 1740, 1783, 1796, and 1873 CE). **e** SEA on the May–August temperature observation target (CRU dataset) using large tropical eruptions (1963 and 1982 CE). **f** Same as in **e**, but using NHET eruptions (1912, 1933, and 1956 CE). The 1933 and 1956 CE eruptions (Volcanic explosivity index = 5) were used to increase number of events, although they had peak $SAOD_{NHET}$ smaller than 0.03. The 0.95 statistical significance level is shown by the horizontal dashed lines in **a**–**d**. The statistical significance is not evaluated in **e** and **f** because only 2–3 eruptions are considered. All tests confirm that tropical eruptions induce more persistent cooling than NHET eruptions.

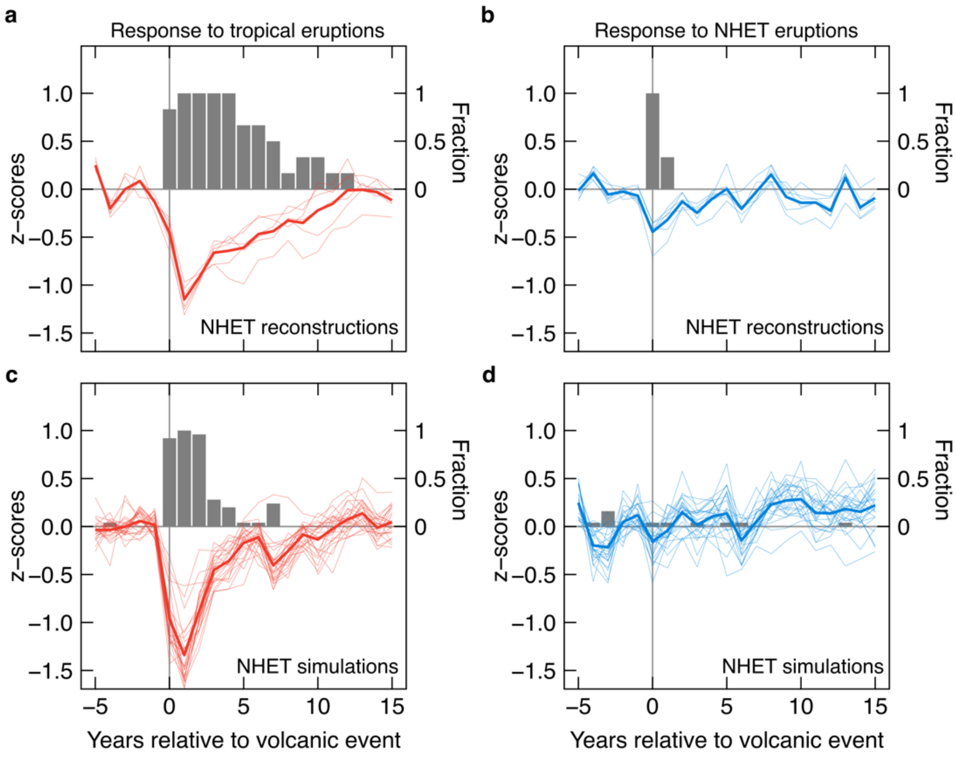

Supplementary Fig. 6. Volcanic responses of Northern Hemisphere extratropical (NHET) tree-ring summer temperature reconstructions (**a, b**) and climate model simulations (**c, d**). The reconstruction and individual CMIP5 simulation time series are the same as those used in Fig. 4 and are transformed to z-scores with respect to the 1000–2000 period prior to superposed epoch analysis (SEA). SEAs are based on all the tropical and NHET eruptions listed in Supplementary Table 7. Thick and thin curves refer to the mean and individual responses, respectively. Bars denote fractions of significant cooling (above the 0.95 statistical significance level) of individual reconstructions or simulations. A fraction of 1 means that all the time series show significant cooling.

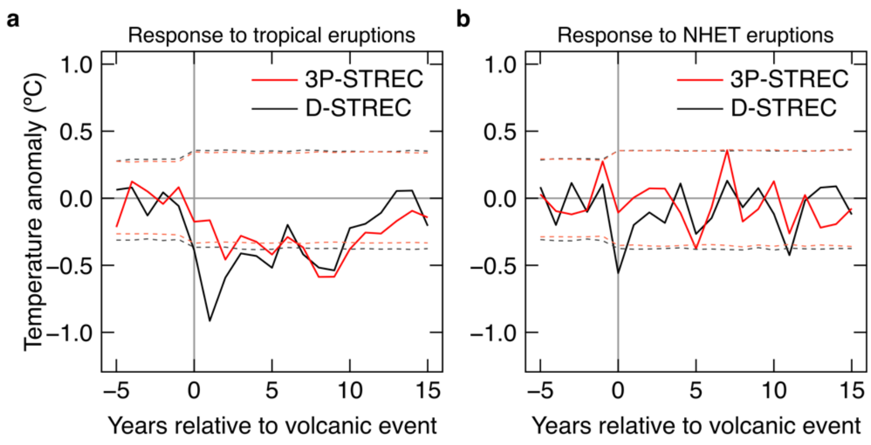

Supplementary Fig. 7. Comparison of volcanic responses between D-STREC and 3P-STREC summer temperature reconstructions in northeastern North America. Superposed epoch analyses are based on all the tropical (**a**) and Northern Hemisphere extratropical (NHET, **b**) eruptions listed in Supplementary Table 7. Horizontal dashed lines refer to the 0.95 statistical significance level for D-STREC (black) and 3P-STREC (red).

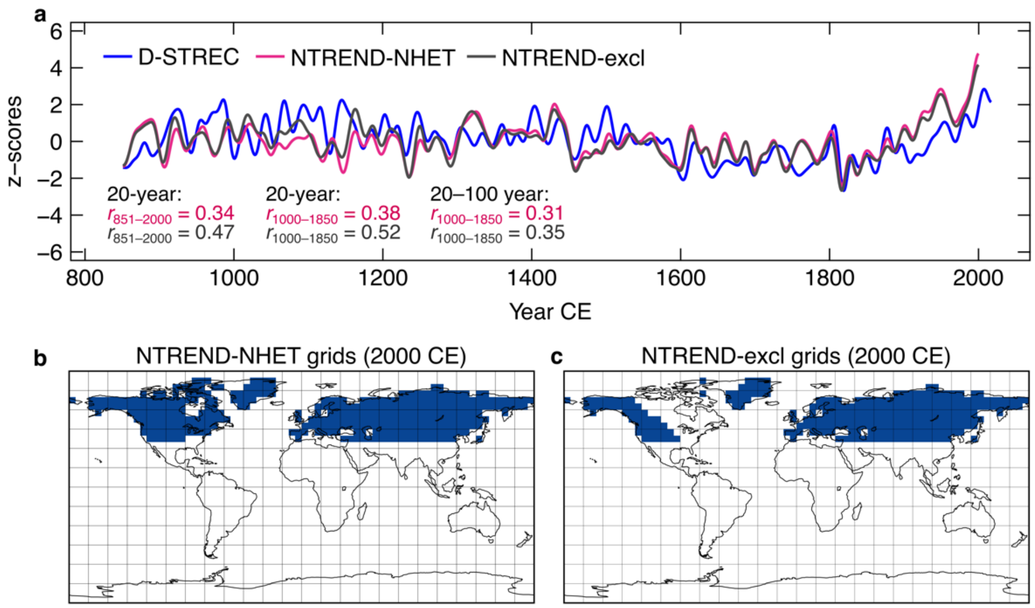

Supplementary Fig. 8. D-STREC compared with Northern Hemisphere extratropical land mean of the gridded NTREND reconstruction[14] (NTREND-NHET) and the hemispheric mean excluding the northeastern North America (NENA) component (NTREND-excl) using the spatial domain of CRU data shown in Fig. 1c. **a** 20-year smoothed time series as well as Pearson's *r* with D-STREC at different time scales. Colored Pearson's *r* corresponds to the legend. In **b** and **c** blue cells indicate the grids used to generate NTREND-NHET and NTREND-excl temperature series at 2000 CE, respectively. Higher correlations with the non-NENA composite than with the whole NTREND-NHET indicate a negligible effect of potential data and geographical overlaps with D-STREC, and emphasize the fact that NENA is underrepresented in hemispheric millennial temperature reconstructions. Maps were generated using the Panoply 5 Software: https://www.giss.nasa.gov/tools/panoply/.

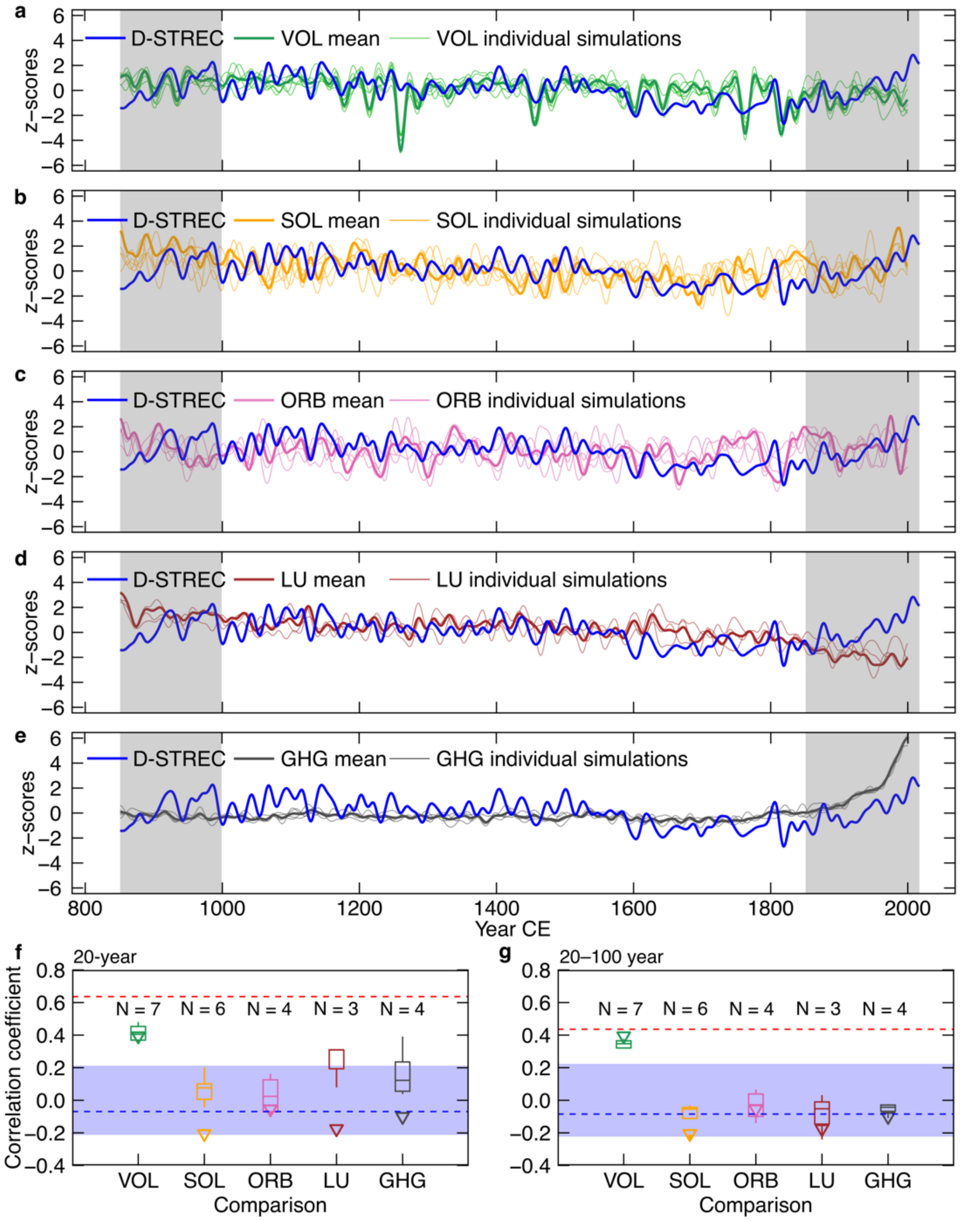

Supplementary Fig. 9. Comparison of D-STREC with single-forcing simulations of Northern Hemisphere extratropical land summer temperatures from the CESM-LME. **a**–**e** D-STREC versus volcanic (VOL), solar (SOL), orbital (ORB), land use (LU), and greenhouse gases (GHG) only simulations. All the time series are smoothed using a 20-year low-pass Butterworth filter and transformed to z-scores with respect to the 1000–2000 time period. **f** Pearson's *r* between the 20-yr smoothed D-STREC and the single-forcing simulations during 1000–1850 CE. Red and blue dashed lines denote the correlations with the full-forcing multi-model mean and the unforced 850 control, respectively. Boxes show the median and the 25%–75% range, whiskers the 1.5 times interquartile range of correlations with (N) individual simulations. Triangles refer to correlations with the mean of simulations. Blue shading shows the 95% confidence interval of correlations among 1000 random red-noise series of D-STREC and the control run (see Methods). **g** Same as **f**, but for the 20–100-year band-pass filtered time series during 1000–1850 CE. The pre-1000 and post-1850 (shaded) periods were excluded for comparison in order to avoid influences of less accurate volcanic forcing in earlier periods and recent anthropogenic warming, respectively.

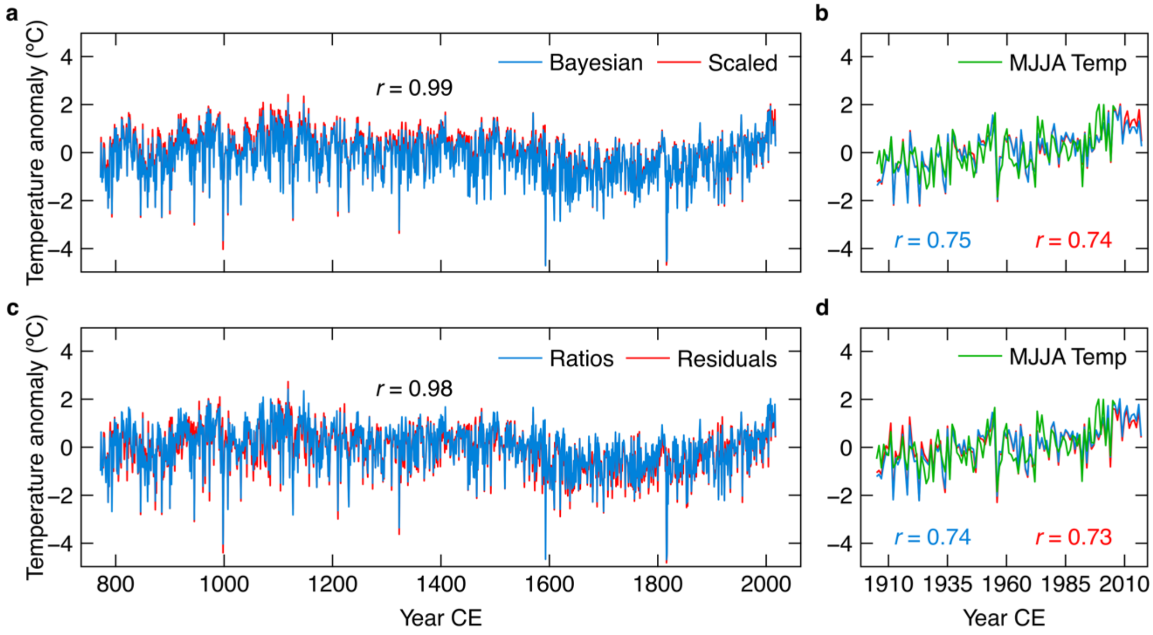

Supplementary Fig. 10. Comparison of temperature reconstruction methods. **a**, **b** Reconstructions using RSFi (regionally constrained individual signal-free standardization) ratio chronologies based on the Bayesian and linear scaling methods. **c**, **d** Reconstructions using RSFi ratio and residual chronologies based on the scaling method. *r* values in **b** and **d** refer to correlations between reconstructions and the regional May–August (MJJA) temperature target over the 1905–2006 time period.

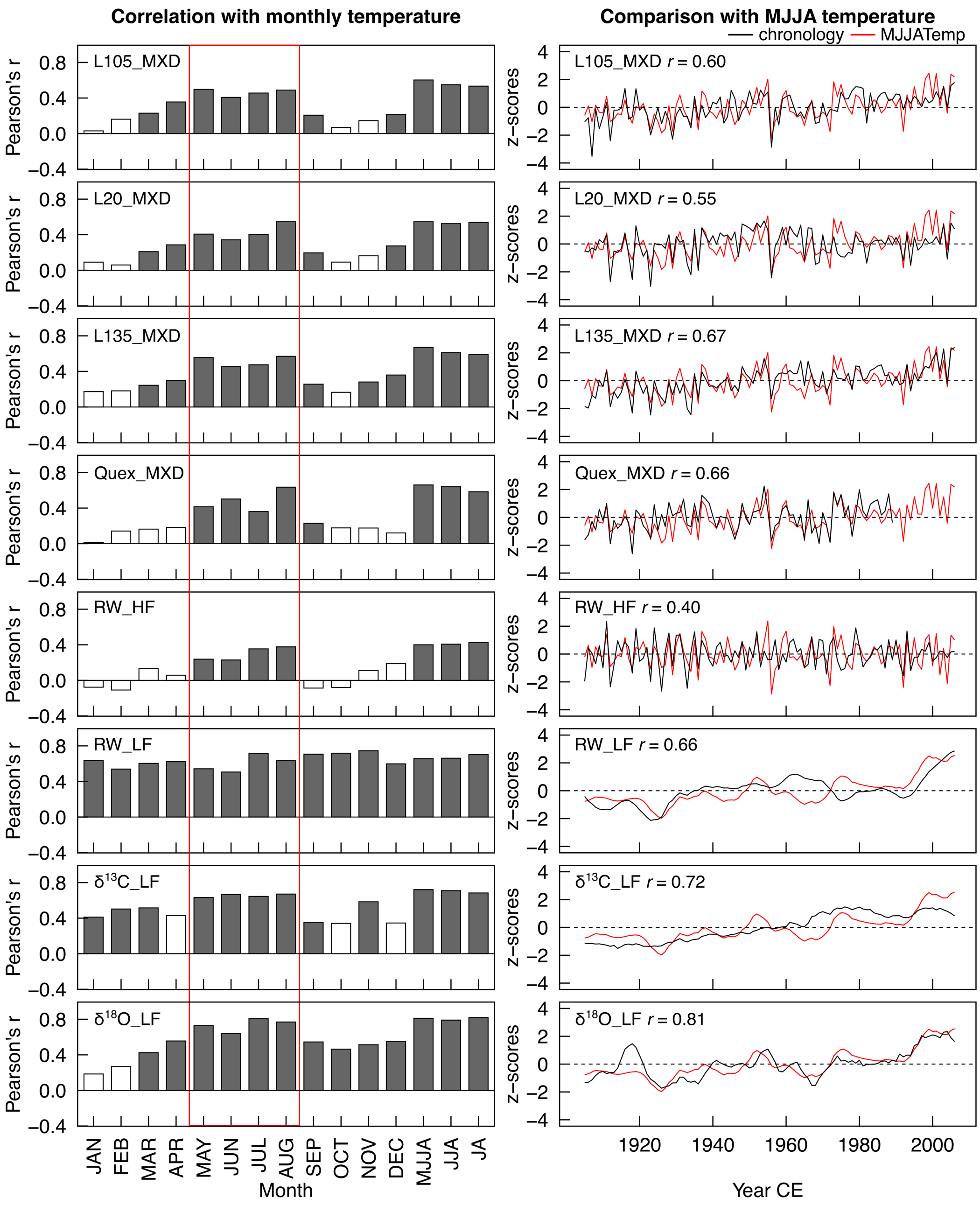

Supplementary Fig. 11. Tree-ring responses to regional mean temperatures from the CRU TS 4.03 dataset over the 1905–2006 time period (1905–1989 CE for maximum latewood density (MXD) at Quex). Left panel: monthly temperature correlations where gray bars denote significant Pearson's correlation coefficients ($P < 0.05$; see Methods). Right panel: comparisons between tree-ring series and May–August (MJJA) regional mean temperatures. The four uppermost and lowermost panels refer to the various components of the D-STREC and 3P-STREC reconstructions, respectively. RW_LF and RW_HF refer to the high- and low-frequency ring width components of 3P-STREC, respectively.

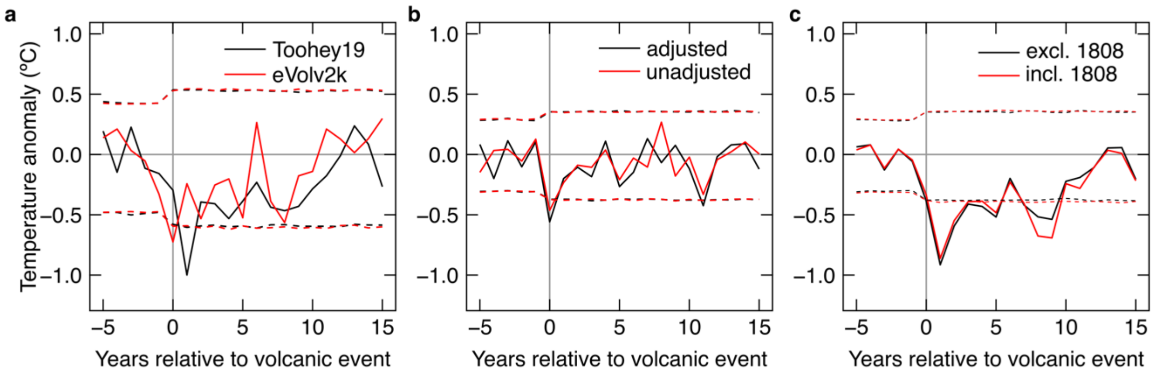

Supplementary Fig. 12. Tests of D-STREC responses to volcanic eruptions. **a** Superposed epoch analysis (SEA) using 10 tropical events with the key years directly derived from eVolv2k[15] (1171, 1191, 1230, 1276, 1286, 1345, 1453, 1458, 1585, and 1695 CE) and adjusted by Toohey19[16] (1170, 1190, 1229, 1275, 1285, 1343, 1452, 1457, 1586, and 1694 CE). **b** SEA using 19 Northern Hemisphere extratropical (NHET) events with adjusted and unadjusted eruption years. A few key years were adjusted according to available eruption months (see the first two columns in Supplementary Table 7). **c** SEA using 24 tropical eruptions with (incl. 1808) and without (excl. 1808) the 1808 CE event.

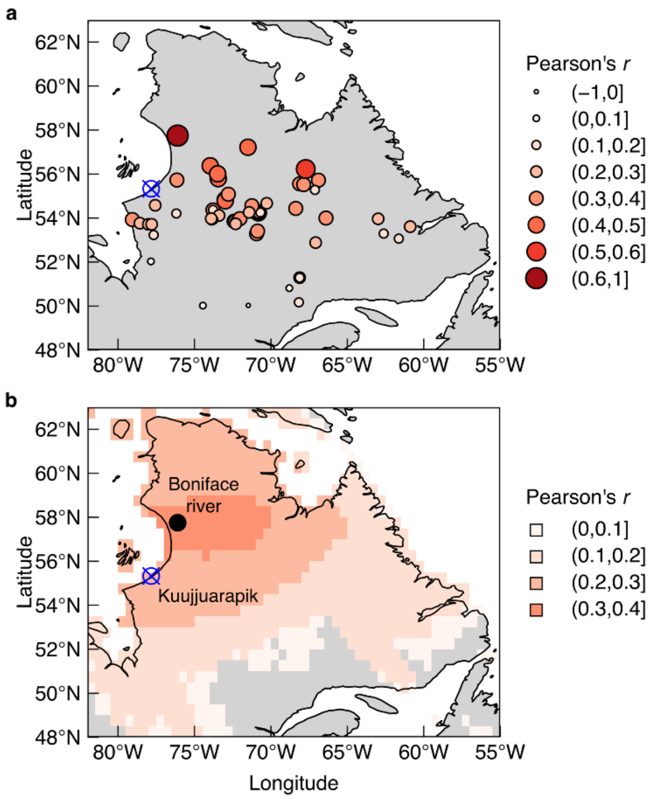

Supplementary Fig. 13. Validation of the location of the Quex tree-ring dataset. **a** Spatial pattern of correlation coefficients between the Quex ring-width chronology and 60 local black spruce chronologies over the 1901–1988 period. Ring-width data were obtained from Wang *et al.*[4], Boucher *et al.*[17], Vallée and Payette[18], and the NOAA International Tree Ring Data Bank. **b** Correlation field of the raw Quex ring-width chronology against April–July 0.5º gridded mean temperatures of the CRU TS 4.03 dataset over the 1901–1988 period. Geographic borderlines are made with Natural Earth. Free vector and raster map data @ naturalearthdata.com.

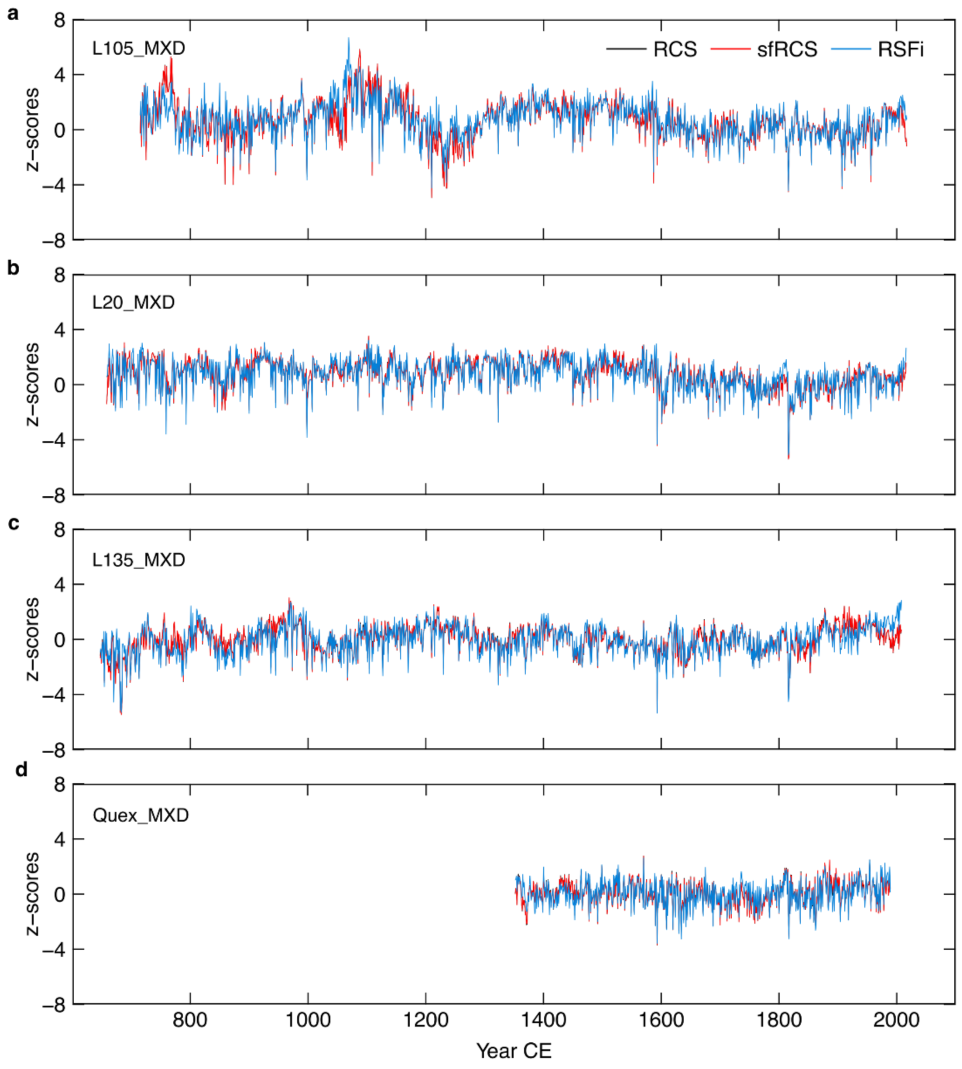

Supplementary Fig. 14. Comparison of standardization methods for maximum latewood density (MXD) ratio chronologies. **a–d** Chronologies for sites L105, L20, L135, and Quex, respectively. RCS: regional curve standardization; sfRCS: signal-free regional curve standardization; RSFi: regionally constrained individual signal-free standardization. All chronologies are transformed to z-scores with respect to the 1601–2000 time period.

Supplementary Tables

Supplementary Table 1. Published millennial maximum latewood density chronologies previously used for climate reconstructions. ITRDB: the NOAA International Tree Ring Data Bank.

Site name	Continent	Lat. / Long. (º)	Species	Timespan CE	Reference
Forfjorddalen	Europe	68.8 / 15.7	*Pinus sylvestris*	800–2005	McCarroll *et al.*[19]
Jämtland		63.3 / 13.3	*Pinus sylvestris*	783–2011	Zhang *et al.*[20]
Laanila		68.5 / 27.3	*Pinus sylvestris*	800–2005	McCarroll *et al.*[19]
Lauenen		46.4 / 7.3	*Picea abies*	982–1976	Schweingruber, ITRDB
Lötschental		46.3 / 7.8	*Larix decidua*	735–2004	Büntgen *et al.*[21]
N-Greece		40.1 / 20.9	*Pinus heldreichii*	730–2015	Esper *et al.*[22]
Nscan		67.5 / 22.5	*Pinus sylvestris*	−215–2006	Esper *et al.*[23]
S-Finland		61.9 / 29.0	*Pinus sylvestris*	674–2000	Helama *et al.*[24]
Torneträsk		68.2 / 19.5	*Pinus sylvestris*	441–2010	Melvin *et al.*[25]
Altai	Asia	50.0 / 88.0	*Larix sibirica*	462–2007	Schneider *et al.*[26]
Polar Ural		66.5 / 65.4	*Larix sibirica*	778–2006	Briffa *et al.*[27]
Athabasca	N. America	52.3 / −117.3	*Picea engelmannii*	869–1994	Luckman and Wilson[28]

Supplementary Table 2. Properties of the maximum latewood density (MXD) network. No. radii / tree: number of measured radii and corresponding trees. MSL: mean segment length of individual MXD series. AR1: mean first-order autocorrelation of raw MXD series. MSL and AR1 are on tree basis. Timespans show periods of the four local MXD chronologies used in D-STREC, whereas bracketed years refer to the start of each chronology. L105, L20, and L135 are three newly sampled sites and the site L135 is composed of three adjacent lakes (L13, L14, and L15, distance < 3km). Quex was developed by Fritz H. Schweingruber, and is available on the NOAA International Tree Ring Data Bank: https://www.ncei.noaa.gov/access/paleo-search/study/4349. Note that the location of Quex has been corrected (Supplementary Method 1).

Site	Lat. (°)	Long. (°)	Elev.(m)	No. radii / tree	MSL	AR1	Timespan CE
L105	50.8	−68.8	531	788 / 647	87	0.56	772 (715) – 2017
L20	54.6	−71.2	483	583 / 438	76	0.41	772 (658) – 2016
L135	56.7	−74.0	251	214 / 164	90	0.40	772 (647) – 2008
Quex	57.8	−76.2	50	83 / 45	134	0.33	1363 (1352) – 1989

Supplementary Table 3. Calibration-validation statistics of summer temperature reconstructions calibrated over the 1905–2006 full period and two sub-intervals. For *r*, the raw, low, and high represent results for unfiltered, 10-year low-pass, and high-pass filtered series, respectively (Butterworth filter). Significant ($P < 0.05$) *r* values are indicated by *. CI: confidence interval of Bayesian reconstructions. 95% CI coverage: percentage of observed temperature values (CRU dataset) within the 95% CI. R^2 was obtained using ordinary-least-square linear regressions between reconstructions and temperature targets. RMSE: root-mean-square error. The continuous potential ranked probability score (CRPSpot) and the reliability score (Reli) are more proper than reduction of error and coefficient of efficiency to assess Bayesian reconstructions[29,30] and were calculated from 1000 members randomly drawn from posteriors of each reconstruction. Validation statistics are indicated by $^{\#}$.

**Calibration period (CE)**	**Statistics**	**D-STREC**			**3P-STREC / 3P-STREC-TRW**		
		**1905–2006**	**1905–1955**	**1956–2006**	**1905–2006**	**1905–1955**	**1956–2006**
1905–2006	*r* (raw)	0.75*	0.72*	0.75*	0.59*/0.53*	0.64*/0.59*	0.50*/0.41*
	r (high)	0.72*	0.69*	0.70*	0.40*/0.40*	0.61*/0.58*	0.22 / 0.28
	r (low)	0.80*	0.79*	0.82*	0.85*/0.73*	0.71*/0.62*	0.93* / 0.58
	95%CI width	1.84	1.79	1.89	3.33 / -	3.33 / -	3.33 / -
	95%CI coverage	87%	82%	92%	98% / -	100% / -	96% / -
	R^2	0.56	0.52	0.57	0.35 / 0.28	0.41 / 0.34	0.25 / 0.17
	RMSE	0.58	0.58	0.58	0.70 / 0.79	0.62 / 0.64	0.77 / 0.91
	CRPSpot	0.33	0.32	0.33	0.39 /-	0.31 / -	0.42 / -
	Reli ($\times10^{-2}$)	1.99	2.70	2.31	0.80e /-	3.66 / -	1.61 / -
1905–1955	R^2	0.54	0.52	$0.53^{\#}$	0.22 / 0.29	0.23 / 0.32	$0.20^{\#}/0.18^{\#}$
	RMSE	0.58	0.56	$0.61^{\#}$	0.76 / 0.82	0.62 / 0.64	$0.87^{\#}/0.96^{\#}$
	CRPSpot	0.33	0.31	$0.34^{\#}$	0.42 / -	0.32 / -	$0.45^{\#}$ / -
	Reli ($\times10^{-2}$)	2.80	2.94	$3.56^{\#}$	2.04 / -	1.30 / -	$9.65^{\#}$ / -
1956–2006	R^2	0.58	$0.52^{\#}$	0.59	0.27 / 0.31	$0.16^{\#}/0.37^{\#}$	0.27 / 0.19
	RMSE	0.60	$0.60^{\#}$	0.59	0.73 / 0.89	$0.71^{\#}/0.83^{\#}$	0.75 / 0.94
	CRPSpot	0.32	$0.31^{\#}$	0.32	0.40 / -	$0.35^{\#}$ / -	0.43 / -
	Reli ($\times10^{-2}$)	3.32	$4.23^{\#}$	3.37	1.85 / -	$4.68^{\#}$ / -	1.38 / -

Note: 3P-STREC-TRW is the tree-ring width (TRW) component of 3P-STREC and is considered here to show the improved reconstruction skill of maximum latewood density data compared to the more commonly used TRW data. This time series was not calibrated using the same Bayesian method as used by D-STREC and 3P-STREC but using the linear scaling approach. Therefore, 95% CI width, 95% CI coverage, CRPSpot, and Reli are not available for the 3P-STREC-TRW.

Supplementary Table 4. Non-overlapping warmest decades and strongest centennial warming trends in D-STREC. Only positive non-overlapping trends are shown. * and #: equal rank. SE: standard error.

Rank	Warmest decades		100-year warming trends	
	**Period**	**Temp. ºC**	**Period**	**ºC /10 y ± SE**
1	2005–2014	1.25	1917–2016	0.16 ± 0.024
2*	1139–1148	0.97	840–939	0.14 ± 0.027
3*	983–992	0.97	998–1097	0.12 ± 0.028
4	1063–1072	0.92	1816–1915	0.09 ± 0.031
5#	1400–1409	0.82	1316–1415	0.07 ± 0.026
6#	966–975	0.82	1640–1739	0.07 ± 0.024
7	1093–1102	0.78	1438–1537	0.05 ± 0.027
8	1111–1120	0.77	1170–1269	0.03 ± 0.027
9	1497–1506	0.75	-	-
10	811–820	0.75	-	-

Supplementary Table 5. Non-overlapping coldest decades reconstructed by D-STREC since 772 CE and correspondence to volcanic eruptions according to the eVolv2k[15] (a), IVI2[31] (b), and ICI[32] (c) eruption datasets. Information on identified eruptions is provided by the Global Volcanism Program (GVP)[33]. The last column shows the locations of matched eruptions, chronologically. T: Tropical eruptions; E: Northern Hemisphere extratropical eruptions. *: events matched by all the three ice-core datasets. UNI: unidentified events. #: matches provided by Toohey *et al.*[15].

**Rank**	**Period CE**	**Temp. ºC**	**Match to volcanic eruptions**	**Location**
1	1816–1825	−1.70	Tambora (1815*); Galunggung (1822 a)	T; T
2	1601–1610	−1.36	Huaynaputina (1600*); UNI (1607 c)	T; T
3	1693–1702	−1.29	Serua (1693*); UNI (1694/1695 a/c)	T; T
4	1640–1649	−1.22	Parker (1640*); Komagatake (1640 c); UNI (1645/1646 c/a)	T; E; E
5	1753–1762	−1.19	Katla (1755 a/b); UNI (1760 c); UNI (1762*)#	E; E; T
6	1629–1638	−1.11	Furnas (1630 c), UNI (1637 a)	E; E
7	1764–1773	−1.06	UNI (1762*)#; Hekla (1766 a); UNI (1770 a); UNI (1772 c)	T; E; E; E
8	1853–1862	−1.05	Toya (1853 a/c); Komagatake (1856 a); Katla (1860 c); Makian (1861*)	E; E; E; T
9	1836–1845	−1.04	Cosiguina (1835*); UNI (1840 c); UNI (1843 c)	T; E; E
10	780–789	−1.02	UNI (782 a)	E

Supplementary Table 6. Extremely cold years (≤ mean−2SD) in the D-STREC reconstruction and correspondence to volcanic eruptions according to the eVolv2k[15], IVI2[31], and ICI[32] eruption datasets (Methods). Information of identified eruptions is provided by the Global Volcanism Program (GVP)[33]. Temperatures are shown in anomalies (ºC) with respect to the 1905–2006 time period. Question marks refer to uncertain matches. #: unidentified events matched by Toohey *et al.*[15]. *: eruption years adjusted by Toohey *et al.*[16]. NHET: Northern Hemisphere extratropical regions. The higher frequency of unmatched cold years to eruptions in the first millennium may be related to less accurate ice-core dating and lack of historical records.

Year CE	Temp. ºC	Volcano	GVP year	Match to volcanic eruptions			Location
				eVolv2k	IVI2	ICI	
786	−2.31	-	-	-	-	No data	-
793	−2.53	-	-	-	-	No data	-
846	−2.60	Churchill?	847 ± 1?	-	-	845	NHET
859	−2.28	Unidentified	-	859/858*	-	-	Tropical
873	−2.28	-	-	-	-	-	-
885	−2.68	-	-	-	-	-	-
900	−2.39	Unidentified	-	900/899*	901?	-	Tropical
945	−2.89	-	-	-	-	-	-
998	−3.66	Unidentified	-	998	-	-	NHET
1007	−2.50	Unidentified	-	-	-	1006	NHET
1031	−2.17	Arenal?	1030	1028/1029*	1030?	-	Tropical
1127	−2.71	Unidentified	-	1127/1125*	-	1128?	Tropical
1210	−2.42	Katla	1210	1210	-	-	NHET
1230	−2.46	Unidentified	-	1230/1229*	1227#	1228#	Tropical
1323	−3.22	-	-	-	-	-	-
1466	−2.13	-	-	-	-	-	-
1480	−1.95	St. Helens	1480	1480	1480	1480	NHET
1492	−1.95	-	-	-	-	-	-
1593	−4.71	Raung	1593	1595/1594*?	1593	1593	Tropical
1609	−2.82	Unidentified	-	-	-	1607	NHET
1620	−2.86	Unidentified	-	-	1619	1620	Tropical
1630	−2.02	Unidentified	-	-	-	1630	NHET
1634	−2.17	-	-	-	-	-	-
1638	−2.46	Unidentified	-	1637	-	-	NHET
1642	−2.06	Parker	1640	1640	1641	1640	Tropical
1644	−1.95	-	-	-	-	-	-
1654	−1.99	Unidentified	-	1654/1653*	-	-	Tropical
1680	−2.24	Tangkoko	1680	-	-	1680	Tropical
1688	−2.42	Unidentified	-	-	-	1686	Tropical
1700	−2.75	Unidentified	-	-	-	1700	NHET
1757	−2.28	Katla	1755	1755	1755	-	NHET
1759	−1.99	-	-	-	-	-	-
1767	−2.06	Hekla	1766	1766	-	-	NHET
1816	−4.53	Tambora	1815	1815	1815	1815	Tropical
1817	−4.42	Tambora	1815	1815	1815	1815	Tropical
1819	−2.50	-	-	-	-	-	-
1836	−2.31	Cosiguina	1835	1835	1835	1835	Tropical
1853	−2.42	Toya	1853	1853	-	-	NHET
1855	−2.24	Sheveluch	1854	1856?	-	1854	NHET
1862	−2.10	Makian	1861	1861	1861	1861	Tropical
1912	−2.13	Novarupta	1912	No data	1912	1912	NHET
1918	−2.10	Unidentified	-	No data	-	1916	NHET
1923	−2.13	-	-	No data	-	-	-
1956	−1.95	Bezymianny	1955	No data	-	1956	NHET

The eVolv2k v3 dataset is available at:

https://cera-www.dkrz.de/WDCC/ui/cerasearch/entry?acronym=eVolv2k_v3.
The IVI2 dataset is available at: http://climate.envsci.rutgers.edu/IVI2/#Version2.
The ICI dataset is available at: https://www.ncei.noaa.gov/access/paleo-search/study/14168.

Supplementary Table 7. Tropical and Northern Hemisphere extratropical (NHET) volcanic eruptions used for superposed epoch analysis (SEA), with peak stratospheric aerosol optical depth at 550 nm over 30–90ºN ($SAOD_{NHET}$) ≥ 0.03 according to the eVolv2k[15] (1000–1900 CE) plus CMIP6[34] (1901–2016 CE) dataset. Volcanic explosivity index (VEI) is provided by the Global Volcanism Program (GVP)[33]. The IVI2[31] and ICI[32] were used to screen unidentified eruptions (Methods). [#]: unidentified events matched by Toohey *et al.*[15]. *: years adjusted by Toohey *et al.*[16]. [a]: years adjusted by authors. [b]: categorized as unidentified events. [c]: years refers to the GVP. ?: uncertain values. In Eruption Year column, eruption months are shown after dots with uncertainty in brackets, e.g., 1257.7 (±3) shows the Samalas erupted around July of 1257 CE with a 3-month uncertainty. Key years were used for SEAs on reconstructions and simulations, while ice years from the same eVolv2k dataset were used for SEAs on $SAOD_{NHET}$ (i.e., Fig. 3c, d).

Key Year CE	Eruption Year	Volcano	VEI	Peak $SAOD_{NHET}$ (Year)	Ice year			Location
					eVolv2k	IVI2	ICI	
1020	1020	Unidentified	-	0.051 (1020)	1020	1018	1021	NHET
1170	1170*	Unidentified	-	0.157 (1172)	1171	1167[#]	1169[#]	Tropical
1180	1180*	Unidentified	-	0.246 (1182)	1182	1176[#]	1184[#]	NHET
1190	1190*	Unidentified	-	0.063 (1192)	1191	1188	1193	Tropical
1210	1210	Katla	4	0.083 (1210)	1210	-	-	NHET
1229	1229*	Unidentified	-	0.212 (1231)	1230	1227[#]	1228[#]	Tropical
1257	1257.7(±3)	Samalas	7?	0.482 (1258)	1257	1258	1257	Tropical
1275	1275*	Unidentified	-	0.047 (1277)	1276	1275[#]	1275[#]	Tropical
1285	1285*	Unidentified	-	0.121 (1287)	1286	1284[#]	1286[#]	Tropical
1328	1328*	Unidentified	-	0.092 (1329)	1329	1328	1329	NHET
1343	1343*	Unidentified	-	0.125 (1346)	1345	1341[#]	1344[#]	Tropical
1452	1452*	Kuwae?[b]	-	0.102 (1454)	1453	1452[#]	1454	Tropical
1457	1457*	Unidentified	-	0.211 (1459)	1458	1459[#]	1456[#]	Tropical
1477	1477.2	Bardarbunga	6	0.118 (1477)	1477	1476	1474	NHET
1480	1480.1(±4)	St. Helens	5+	0.052 (1480)	1480	1480	1480	NHET
1510	1510.7	Hekla	4	0.051 (1511)	1510	1512	1508	NHET
1586	1586*	Kelud	5?	0.092 (1586)	1585	1584	1585?	Tropical
1593	1593[a]	Raung	5?	0.075 (1596)	1595	1593	1593	Tropical
1600	1600.2	Huaynaputina	6	0.182 (1601)	1600	1600	1600	Tropical
1641	1640.12	Parker	5?	0.163 (1640)	1640	1641	1640	Tropical
1668	1667.9	Shikotsu	5	0.085 (1668)	1667	-	1667	NHET
1673	1673.5	Gamkonora	5?	0.043 (1674)	1673	1673	1673	Tropical
1694	1694*	Unidentified	-	0.126 (1696)	1695	1693[#]	1694[#]	Tropical
1708	1707.12	Fujisan	5	0.031 (1708)	1707	-	1704	NHET
1730	1730*	Unidentified	-	0.119 (1729)	1729	1729[#]	1731[#]	NHET
1740	1739.8	Shikotsu	5	0.080 (1740)	1739	-	1739	NHET
1756	1755.10	Katla	5?	0.032 (1756)	1755	1755	-	NHET
1762	1762	Unidentified	-	0.044 (1763)	1762	1761[#]	1762[#]	Tropical
1766	1766.4	Hekla	4	0.050 (1766)	1766	-	-	NHET
1783	1783.5	Grimsvotn	4+	0.378 (1784)	1783	1783	1783	NHET
1796	1796[a]	Unidentified	-	0.030 (1797)	1797	1796[#]	1796[#]	NHET
1815	1815.4	Tambora	7	0.250 (1816)	1815	1815	1815	Tropical
1831	1831	Babuyan Claro	4?	0.137 (1832)	1831	1831	1831	Tropical
1835	1835.1	Cosiguina	5	0.087 (1836)	1835	1835	1835	Tropical
1857	1856.9	Komagatake	4	0.034 (1857)	1856	-	1854	NHET
1862	1861.12	Makian	4?	0.041 (1862)	1861	1861	1861	Tropical
1873	1873.1	Grimsvotn	4	0.031 (1873)	1873	-	1872	NHET
1883	1883.5	Krakatau	6	0.116 (1884)	1883	1883	1883	Tropical
1903	1902.10	Santa Maria	6?	0.108 (1903)	No data[c]	1902	1902	Tropical
1907	1907.3	Ksudach	5	0.034 (1907)	No data[c]	-	1907	NHET
1912	1912.6	Novarupta	6	0.103 (1912)	No data[c]	1912	1912	NHET
1963	1963.2	Agung	5	0.030 (1965)	No data[c]	1963	1963	Tropical
1982	1982.3	Chichon, El	5	0.096 (1983)	No data[c]	1982	1982	Tropical

Note: Four events were discarded for SEA based on case-specific reasons. Aira (1471 CE): the eruption likely lasted for several years. Serua (1693 CE): there is only a one-year lag to the stronger 1694 eruption. Unidentified (1808 CE according to the GVP): strong interference with the Tambora eruption (1815 CE; Supplementary Fig. 12c). Pinatubo (1991 CE): too strong influence of the recent warming against the volcanic cooling trend.

Supplementary Table 8. Correlations among multidecadal D-STREC and Northern Hemisphere extratropical (NHET) summer temperature reconstructions and simulations. Refer to Fig. 4 for abbreviations of reconstructions. For comparisons of reconstructions with CMIP5 simulations (NHET sim), correlation coefficients refer to the multi-model mean (unbracketed) and range of the 25 ensemble members (in squared bracket). Simulated northeastern North American temperatures (NENA sim) were averaged from the dotted squared area in Fig. 1a for a direct data-model comparison. ns: number of non-significant (*P* > 0.05; Methods) correlations with single simulations.

**Compared group**	**20-yr low-pass 851–2000 CE**	**20-yr low-pass 1000–1850 CE**	**20–100 yr band-pass 1000–1850 CE**
D-STREC vs. Guillet20	0.39	0.43	0.46
D-STREC vs. NTREND-all	0.56	0.58	0.49
D-STREC vs. NTREND-D	0.55	0.67	0.41
D-STREC vs. NTREND-excl	0.47	0.52	0.35
D-STREC vs. Sch 15	0.47	0.50	0.43
D-STREC vs. Sto15	0.52	0.56	0.48
D-STREC vs. NHET sim	0.55 [0.15–0.60, ns: 1]	0.64 [0.19–0.63, ns: 1]	0.44 [0.21–0.45, ns: 1]
D-STREC vs. NENA sim	0.40 [0.02–0.40, ns: 6]	0.62 [0.10–0.50, ns: 6]	0.41 [−0.14–0.37, ns: 17]
Guillet20 vs. NHET sim	0.46 [0.22–0.51, ns: 0]	0.35 [0.10–0.43, ns: 5]	0.46 [0.13–0.56, ns: 2]
NTREND-all vs. NHET sim	0.64 [0.36–0.66, ns: 0]	0.56 [0.13–0.68, ns: 1]	0.32 [0.01–0.55, ns: 13]
NTREND-D vs. NHET sim	0.66 [0.35–0.66, ns: 0]	0.60 [0.17–0.65, ns: 1]	0.42 [0.06–0.62, ns: 5]
NTREND-excl vs. NHET sim	0.61 [0.33–0.73, ns: 0]	0.48 [0.14–0.63, ns: 2]	0.27 [0.02–0.56, ns: 19]
Sch15 vs. NHET sim	0.50 [0.27–0.50, ns: 0]	0.40 [0.07–0.49, ns: 2]	0.43 [0.17–0.63, ns: 4]
Sto15 vs. NHET sim	0.63 [0.37–0.61, ns: 0]	0.62 [0.24–0.66, ns: 0]	0.56 [0.22–0.66, ns: 1]
NENA sim vs. NHET sim	[0.42–0.85, ns: 0]	[0.36–0.82, ns: 0]	[0.14–0.80, ns: 1]

Note: Multidecadal coherence of D-STREC with NENA simulations is not as strong as that with NHET simulations, as regional simulations are prone to a higher level of modeled internal variability.

Supplementary Table 9. CMIP5 last-millennium ensemble simulations and volcanic forcing datasets used. GRA: Gao *et al.*[31]; CEA: Crowley *et al.*[35]; AJS: Ammann *et al.*[36] . FULL: full-forcing; GHG: greenhouse gases only; LU: land use only; ORB: orbital only; SOL: solar only; VOL: volcanic only simulations; CNTL: unforced control run. Note that the full-forcing members of the CESM-LME and CMIP5 (Past1000 stitched to the historical experiments) simulations were named CMIP5 simulations in the main text.

**Sub-ensemble**	**Model**	**Type**	**Volcanic forcing**	**Member**	**Reference**
CESM-LME	CESM-CAM5	FULL	GRA	1–13	Otto-Bliesner *et al.*[37]
		GHG	-	1–3	
		LU	-	1–3	
		ORB	-	1–3	
		SOL	-	1, 3–5	
		VOL	GRA	1–5	
		CNTL	-	1	
	iCESM1	FULL	GRA	1–3	Brady *et al.*[38]
		GHG	-	1	
		ORB	-	1	
		SOL	-	1	
		VOL	GRA	1–2	
CMIP5 Past1000 + historical experiments	BCC-CSM1.1	FULL	GRA	1	Xin *et al.*[39]
	CCSM4	FULL	GRA	1	Landrum *et al.*[40]
	CSIRO-Mk-3L-1-2	FULL	GRA	1	Phipps *et al.*[41]
	GISS-E2-R	FULL	CEA & GRA	r121 & r128	Schmidt *et al.*[42]
	IPSL-CM5A-LR	FULL	AJS	1	Dufresne *et al.*[43]
	MPI-ESM-P	FULL	CEA	1	Jungclaus *et al.*[44]
	MRI-CGCM3	FULL	GRA	1	Yukimoto *et al.*[45]
	HadCM3	FULL	CEA	1	Schurer *et al.*[46]

CESM-LME simulations are available at: https://www.cesm.ucar.edu/projects/community-projects/LME/data-sets.html.
CMIP5 Past1000 and historical simulations are available at: https://esgf-node.llnl.gov/projects/esgf-llnl/.

Supplementary Table 10. Correlations among sites and May–August (MJJA) temperatures according to standardization methods. PT: power transformation. Correlation coefficients were calculated over the common time periods of each pair of time series. Temp-raw and Temp-LP refer to correlations with corresponding unfiltered and 10-year low-pass filtered local CRU MJJA mean temperatures starting from 1901 CE, respectively. RCS: regional curve standardization; sfRCS: signal-free regional curve standardization; RSFi: regionally constrained individual signal-free standardization.

**Standardization Method**		**Ratios**				**Residuals + PT**			
		L105	L20	L135	Quex	L105	L20	L135	Quex
RCS	L20	0.45	-	-	-	0.45	-	-	-
	L135	0.14	0.53	-	-	0.14	0.52	-	-
	Quex	0.18	0.49	0.64	-	0.22	0.53	0.64	-
	Temp-raw	0.59	0.48	0.22	0.59	0.59	0.47	0.21	0.59
	Temp-LF	0.61	0.20	−0.34	0.26	0.60	0.17	−0.36	0.23
sfRCS	L20	0.45	-	-		0.45	-	-	-
	L135	0.14	0.52	-		0.14	0.52	-	-
	Quex	0.17	0.48	0.65		0.21	0.52	0.66	-
	Temp-raw	0.59	0.48	0.22	0.57	0.59	0.47	0.21	0.55
	Temp-LF	0.61	0.20	−0.35	0.16	0.60	0.17	−0.36	0.04
RSFi	L20	0.50	-	-	-	0.55	-	-	-
	L135	0.19	0.53	-	-	0.18	0.50	-	-
	Quex	0.17	0.50	0.62	-	0.19	0.50	0.63	-
	Temp-raw	0.65	0.59	0.68	0.62	0.65	0.60	0.63	0.63
	Temp-LF	0.78	0.55	0.76	0.40	0.78	0.62	0.67	0.41

Supplementary References

1. Payette, S., Filion, L., Gauthier, L. & Boutin, Y. Secular climate change in old-growth tree-line vegetation of northern Quebec. *Nature* **315**, 135–138 (1985).
2. Payette, S., Filion, L., Delwaide, A. & Bégin, C. Reconstruction of tree-line vegetation response to long-term climate change. *Nature* **341**, 429–432 (1989).
3. Frank, D. & Esper, J. Characterization and climate response patterns of a high-elevation, multi-species tree-ring network in the European Alps. *Dendrochronologia* **22**, 107–121 (2005).
4. Wang, F. *et al.* Temperature sensitivity of blue intensity, maximum latewood density, and ring width data of living black spruce trees in the eastern Canadian taiga. *Dendrochronologia* **64**, 125771 (2020).
5. Briffa, K. R. *et al.* Fennoscandian summers from AD 500: temperature changes on short and long timescales. *Clim. Dyn.* **7**, 111–119 (1992).
6. Melvin, T. M. & Briffa, K. R. CRUST: Software for the implementation of regional chronology standardisation: Part 1. Signal-Free RCS. *Dendrochronologia* **32**, 7–20 (2014).
7. Björklund, J. A. *et al.* Advances towards improved low-frequency tree-ring reconstructions, using an updated Pinus sylvestris L. MXD network from the Scandinavian Mountains. *Theor. Appl. Climatol.* **113**, 697–710 (2013).
8. Melvin, T. M., Briffa, K. R., Nicolussi, K. & Grabner, M. Time-varying-response smoothing. *Dendrochronologia* **25**, 65–69 (2007).
9. Osborn, T. J., Biffa, K. & Jones, P. Adjusting variance for sample-size in tree-ring chronologies and other regional-mean timeseries. *Dendrochronologia* **15**, 89–99 (1997).
10. Frank, D. C., Esper, J. & Cook, E. R. On Variance adjustments in tree-ring chronology development. *TRACE–Tree Rings Archaeol. Climatol. Ecol.* 56–66 (2007).
11. Esper, J., Frank, D. C., Wilson, R. J. S. & Briffa, K. R. Effect of scaling and regression on reconstructed temperature amplitude for the past millennium. *Geophys. Res. Lett.* **32**, L07711 (2005).
12. Gennaretti, F. *et al.* Bayesian multiproxy temperature reconstruction with black spruce ring widths and stable isotopes from the northern Quebec taiga. *Clim. Dyn.* **49**, 4107–4119 (2017).
13. Wigley, T. M. L., Briffa, K. R. & Jones, P. D. On the average value of correlated time series, with applications in dendroclimatology and hydrometeorology. *J. Clim. Appl. Meteor.* **23**, 201–213 (1984).
14. Anchukaitis, K. J. *et al.* Last millennium Northern Hemisphere summer temperatures from tree rings: Part II, spatially resolved reconstructions. *Quat. Sci. Rev.* **163**, 1–22 (2017).
15. Toohey, M. & Sigl, M. Volcanic stratospheric sulfur injections and aerosol optical depth from 500 BCE to 1900 CE. *Earth Syst. Sci. Data* **9**, 809–831 (2017).
16. Toohey, M. *et al.* Disproportionately strong climate forcing from extratropical explosive volcanic eruptions. *Nature Geosci.* **12**, 100–107 (2019).
17. Boucher, É., Nicault, A., Arseneault, D., Bégin, Y. & Karami, M. P. Decadal variations in eastern Canada's taiga wood biomass production forced by ocean-atmosphere interactions. *Sci. Rep.* **7**, 2457 (2017).
18. Vallée, S. & Payette, S. Contrasted growth of black spruce (*Picea mariana*) forest trees at treeline associated with climate change over the last 400 years. *Arct. Antarct. Alp. Res.* **36**, 400–406 (2004).
19. McCarroll, D. *et al.* A 1200-year multiproxy record of tree growth and summer temperature at the northern pine forest limit of Europe. *The Holocene* **23**, 471–484 (2013).
20. Zhang, P., Linderholm, H. W., Gunnarson, B. E., Björklund, J. & Chen, D. 1200 years of warm-season temperature variability in central Scandinavia inferred from tree-ring density. *Clim. Past* **12**, 1297–1312 (2016).

21. Büntgen, U., Frank, D. C., Nievergelt, D. & Esper, J. Summer temperature variations in the European Alps, A.D. 755–2004. *J. Clim.* **19**, 5606–5623 (2006).
22. Esper, J. *et al.* Eastern Mediterranean summer temperatures since 730 CE from Mt. Smolikas tree-ring densities. *Clim. Dyn.* **54**, 1367–1382 (2020).
23. Esper, J. *et al.* Orbital forcing of tree-ring data. *Nat. Clim. Change* **2**, 862–866 (2012).
24. Helama, S. *et al.* A palaeotemperature record for the Finnish Lakeland based on microdensitometric variations in tree rings. *Geochronometria* **41**, 265–277 (2014).
25. Melvin, T. M., Grudd, H. & Briffa, K. R. Potential bias in 'updating' tree-ring chronologies using regional curve standardisation: Re-processing 1500 years of Torneträsk density and ring-width data. *The Holocene* **23**, 364–373 (2013).
26. Schneider, L. *et al.* Revising midlatitude summer temperatures back to A.D. 600 based on a wood density network. *Geophys. Res. Lett.* **42**, 4556–4562 (2015).
27. Briffa, K. R. *et al.* Reassessing the evidence for tree-growth and inferred temperature change during the Common Era in Yamalia, northwest Siberia. *Quat. Sci. Rev.* **72**, 83–107 (2013).
28. Luckman, B. H. & Wilson, R. J. S. Summer temperatures in the Canadian Rockies during the last millennium: a revised record. *Clim. Dyn.* **24**, 131–144 (2005).
29. Gneiting, T. & Raftery, A. E. Strictly proper scoring rules, prediction, and estimation. *J. Am. Stat. Assoc.* **102**, 359–378 (2007).
30. Werner, J. P., Divine, D. V., Charpentier Ljungqvist, F., Nilsen, T. & Francus, P. Spatio-temporal variability of Arctic summer temperatures over the past 2 millennia. *Clim. Past* **14**, 527–557 (2018).
31. Gao, C., Robock, A. & Ammann, C. Volcanic forcing of climate over the past 1500 years: An improved ice core-based index for climate models. *J. Geophys. Res. Atmospheres* **113**, D23111 (2008).
32. Crowley, T. J. & Unterman, M. B. Technical details concerning development of a 1200 yr proxy index for global volcanism. *Earth Syst. Sci. Data* **5**, 187–197 (2013).
33. Global Volcanism Program. *Volcanoes of the World, v. 4.8.8*. Venzke, E (ed.) *Smithsonian Institution*. https://doi.org/10.5479/si.GVP.VOTW4-2013 (2013). [Last access: 22 May, 2022].
34. Luo, B. Stratospheric aerosol radiative forcing and SAD version v4 1850–2016. ftp://iacftp.ethz.ch/pub_read/luo/CMIP6_SAD_radForcing_v4.0.0/ (2018) [Last access: 7 July, 2021].
35. Crowley, T. J. *et al.* Volcanism and the Little Ice Age. *PAGES News* 22–23 (2008).
36. Ammann, C. M., Meehl, G. A., Washington, W. M. & Zender, C. S. A monthly and latitudinally varying volcanic forcing dataset in simulations of 20th century climate. *Geophys. Res. Lett.* **30**, (2003).
37. Otto-Bliesner, B. L. *et al.* Climate variability and change since 850 CE: an ensemble approach with the Community Earth System Model. *Bull. Amer. Meteor. Soc.* **97**, 735–754 (2016).
38. Brady, E. *et al.* The connected isotopic water cycle in the Community Earth System Model version 1. *J. Adv. Model. Earth Syst.* **11**, 2547–2566 (2019).
39. Xin, X., Wu, T. & Zhang, J. Introduction of CMIP5 Experiments carried out with the Climate System Models of Beijing Climate Center. *Adv. Clim. Change Res.* **4**, 41–49 (2013).
40. Landrum, L. *et al.* Last millennium climate and its variability in CCSM4. *J. Clim.* **26**, 1085–1111 (2013).
41. Phipps, S. J. *et al.* Paleoclimate data–model comparison and the role of climate forcings over the past 1500 years. *J. Clim.* **26**, 6915–6936 (2013).
42. Schmidt, G. A. *et al.* Present-day atmospheric simulations using GISS modelE: comparison to in situ, satellite, and reanalysis data. *J. Clim.* **19**, 153–192 (2006).
43. Dufresne, J.-L. *et al.* Climate change projections using the IPSL-CM5 Earth System Model: from CMIP3 to CMIP5. *Clim. Dyn.* **40**, 2123–2165 (2013).

44. Jungclaus, J. H. *et al.* Climate and carbon-cycle variability over the last millennium. *Clim. Past* **6**, 723–737 (2010).
45. Yukimoto, S. *et al.* A new global climate model of the Meteorological Research Institute: MRI-CGCM3 —model description and basic performance. *J. Meteorol. Soc. Jpn. Ser II* **90A**, 23–64 (2012).
46. Schurer, A. P., Hegerl, G. C., Mann, M. E., Tett, S. F. B. & Phipps, S. J. Separating forced from chaotic climate variability over the past millennium. *J. Clim.* **26**, 6954–6973 (2013).
